# Medical invalidation is associated with structural barriers in postacute immune mediated syndromes based on patient perspectives in Germany

**DOI:** 10.1038/s41598-026-67883-2

**Published:** 2026-08-25

**Authors:** Jad Saad, Jens Hensen, Corinna Bergelt, Seraina Petra Lerch

**Affiliations:** Department of Medical Psychology, University Medicine Greifswald, Walther-Rathenau-Str. 48, Greifswald, 17475 Germany

**Keywords:** Post-acute immune-mediated syndrome, Post-Acute-infection syndrome, Long Covid, Myalgic Encephalomyelitis/Chronic Fatigue Syndrome, Post-acute COVID-19 vaccination syndrome, Medical invalidation, Healthcare access, Diseases, Health care, Medical research

## Abstract

Post-acute immune-mediated syndromes (PAIMS), including Long COVID/Post-COVID (LC/PC), Myalgic Encephalomyelitis/Chronic Fatigue Syndrome (ME/CFS), and post-acute COVID-19 vaccination syndrome (PACVS), are characterized by persistent, multisystemic symptoms and significant healthcare challenges. A key issue is medical invalidation, defined as the dismissal or delegitimization of patients’ reports of symptoms. This cross-sectional online study, conducted in Germany between October and December 2025 (*N* = 577), examined healthcare experiences, access barriers, and perceived invalidation. Although healthcare utilization was high, 87% of participants reported difficulties accessing appropriate treatment. Major barriers included lack of available therapies (60%) or not being taken seriously (26%). Perceived invalidation by medical personnel was moderate across all groups (LC: M = 2.80; ME/CFS: M = 2.99; PACVS: M = 3.03) and did not differ significantly (Welch-F(2, 190.50) = 2.87, *p* = 0.059) between groups. Invalidation occurred not only within healthcare settings but was particularly pronounced in interactions with authorities and workplaces. Social media and online communities served as important sources of information and support, especially among individuals with ME/CFS. Overall, the findings reveal substantial structural barriers in the care of PAIMS and highlight the need for improved clinical education, better care coordination, and stronger institutional recognition of these conditions.

**Supplementary Information:**

The online version contains supplementary material available at 10.1038/s41598-026-67883-2.

## Introduction

### Background

The COVID-19 pandemic, caused by the global spread of SARS-CoV-2, has resulted not only in acute respiratory disease but also in a range of long-term health sequelae. These include post-acute sequelae of SARS-CoV-2 infection (PASC) and post-acute COVID-19 vaccination syndrome (PACVS). PASC refers to persistent, multisystemic symptoms that arise following acute SARS-CoV-2 infection and may last for months or years^1^. It encompasses conditions such as Long COVID and Post-COVID (LC/PC), which are characterized as heterogeneous multisystem disorders with more than 200 reported symptoms and an estimated prevalence of approximately 10% among infected individuals^2^. LC is typically defined by symptom persistence beyond four weeks after infection, whereas PC describes symptoms lasting longer than twelve weeks^2^. A substantial proportion of individuals with PASC meet diagnostic criteria for Myalgic Encephalomyelitis/Chronic Fatigue Syndrome (ME/CFS), a chronic multisystemic illness that frequently develops following infection^3^. Approximately 4.5% of individuals infected with SARS-CoV-2 fulfill ME/CFS criteria, while 88.7% of ME/CFS cases meet the criteria for LC^3^. Core symptoms shared across these conditions include post-exertional malaise, persistent fatigue, and cognitive impairment^3,4^. Pathophysiologically, ME/CFS and LC/PC exhibit notable overlap, particularly in terms of mitochondrial dysfunction, impaired energy metabolism, and neurobiological alterations. However, neuroimaging findings suggest partially distinct mechanisms, with magnetic resonance spectroscopy studies demonstrating elevated brain lactate levels in ME/CFS and reduced choline concentrations in LC/PC^5^.

PACVS has been described as a chronic condition following SARS-CoV-2 vaccination, characterized by a broad spectrum of symptoms affecting multiple organ systems^6^. Its estimated prevalence is approximately 0.02% among vaccinated individuals. The most commonly reported symptoms include fatigue, generalized malaise, and cognitive dysfunction, and a considerable proportion of affected individuals also meet diagnostic criteria for ME/CFS^6^.

Overall, there is substantial symptomatic and immunological overlap among LC/PC, ME/CFS, and PACVS, despite some differences in clinical presentation and underlying biological mechanisms^7^. As all three conditions are thought to involve, to varying degrees, immune dysregulation and represent chronic sequelae following an acute triggering event^7,8^, no established umbrella term currently encompasses these clinically related conditions. We therefore propose the conceptual umbrella term postacute immune mediated syndromes following pathogen or antigen exposure for conditions associated with the COVID-19 pandemic (PAIMS) for the purpose of this study. This term is intended as a descriptive framework rather than an established disease classification or a statement of a common etiology. To date, therapeutic approaches remain largely limited to symptom-oriented management, and many affected individuals report low expectations of full recovery while attempting to maintain daily functioning^3,9,10^.

Prior to the COVID-19 pandemic, individuals with ME/CFS and other post-infectious conditions already reported substantial deficits in healthcare, including delayed diagnosis, limited access to appropriate treatment, and lack of symptom recognition^11,12^. Despite the subsequent expansion of specialized outpatient services in Germany, many patients continue to report persistent difficulties in accessing adequate diagnosis and care^13^. A key component of effective healthcare is the extent to which patients feel their symptoms are acknowledged. The lack of this acknowledgment is part of the concept of medical invalidation (MI), also referred to as medical gaslighting^14,15^.

MI describes the delegitimization or dismissal of individuals’ illness experiences, including the devaluation of physical symptoms and their attribution to psychological causes. This may manifest as patient-perceived gaslighting or symptom trivialization^16,17^. Closely linked to stigma^15,18^ MI has been widely documented in post-infectious conditions such as LC/PC, ME/CFS, and PACVS. Quantitative studies report high levels of stigmatization and symptom trivialization, which are associated with adverse health outcomes, including reduced social support and increased psychological distress^19,20^. Qualitative research further suggests that MI reflects not merely interpersonal communication failures but also entrenched structural and epistemic biases, particularly skepticism toward subjective symptom reporting and the psychologization of medically unexplained conditions^21^.

MI has thus been identified as a significant barrier to healthcare, often attributed to insufficient clinical knowledge among providers. While much of the existing research is quantitative, the Illness Invalidation Inventory (3*I) enables systematic assessment of perceived invalidation across multiple social domains, including healthcare, family, workplace, and institutional contexts^17,22^. This broader perspective highlights that invalidation is not limited to medical interactions. For example, individuals with ME/CFS report workplace-related stigma, emotional strain, and limited institutional support, often leading to feelings of not being taken seriously^23^. Similarly, studies across chronic conditions, including fibromyalgia and rheumatoid arthritis, consistently show that the strongest experiences of invalidation occur in interactions with social services and authorities^22,24^.

In their search for better medical care and places where they are taken seriously, those affected often organize themselves into online communities used to exchange information with other patients. They share information about treatment options and experiences in dealing with their symptoms^21^.

### Aims of the study

The aim of this study was to investigate patient-reported healthcare experiences, including diagnosis, treatment, MI, and online community use in diseases associated with the COVID-19 pandemic. Our research questions were:

1. Which healthcare professionals are involved in the diagnosis and treatment of PACS and PACVS, and how do healthcare utilization patterns, perceived barriers, and satisfaction differ between the two groups?
2. How often and to what extent do those affected experience medical invalidation, particularly in contact with Health Care Professionals (HCPs), family and relatives, authorities, and in the work context? Do affected individuals experience differences in perceived invalidation in their contact with different groups?
3. Are there differences in social network use and extent of social network use between individuals affected by PACS or PACVS?

## Methods

### Study design

The study was conducted as cross-sectional online survey (Appendix 1) between October and December 2025 using the software REDCap. The study was approved by the Ethics Committee of the University Medical Center Greifswald on August 11, 2025 (Ref. BB 158/25). The survey has been carried out in accordance with the principles of the Declaration of Helsinki. Informed consent was obtained from all participants at the beginning of the survey.

Eligible participants were adults (≥ 18 years) with either suspected or medically confirmed LC, PC or ME/CFS following SARS-CoV-2 infection, or with medically confirmed or suspected health impairments after COVID-19 vaccination. Eligibility criteria were assessed via self-report. Participation required informed consent and agreement to data protection regulations.

Participants were recruited through an online survey (REDCap) distributed via social media platforms (Instagram, Facebook), self-help group networks, and email contacts from a previous qualitative study about healthcare experiences across different chronic and acute diseases. A detailed overview of contacted organizations and outreach is provided in Appendix 2. No financial incentives or compensation were offered.

### Questionnaire

The questionnaire was developed based on an extensive literature review and covered demographics, physical and mental well-being, health status and treatments, perceived healthcare experiences (including MI), and engagement with online community use.

Questionnaires and questionnaire items, which were originally in English, were translated into German using the forward and back translation method. The procedure follows established guidelines for the cross-cultural adaptation of questionnaires^25^. After completion of the development phase, the instrument was pilot-tested with three affected individuals. The comprehensibility, relevance, completeness, practicability, and processing time of the questions were examined. Based on the feedback, typical symptoms were added and minor adjustments were made to the wording. Before finalization, the questionnaire was reviewed by two independent individuals not involved in the study design for comprehensibility and linguistic clarity.

#### Demographics

The questionnaire initially included questions on the sociodemographic characteristics of the participants, including age, gender, marital status, educational level, and employment status before and after the onset of the disease.

#### Health problems and therapy

To inquire about health problems and therapeutic measures, we used questions from the Rehabilitation Needs Questionnaire (RehabNeQ)^26^. These questions cover the perceived health impairments caused by the disease, as well as the therapeutic measures taken and satisfaction with those measures.

#### The care situation from the perspective of those affected

To assess the care situation from the patients’ perspective, we used questionnaire items from Heidelberg University Hospital^27^ and a UK study on LC healthcare experiences^28^. These questionnaires provided us with information on whether the patients’ symptoms were taken seriously^27^, whether there was a tendency to attribute their complaints to psychological causes^28^, and whether all symptoms were adequately addressed during treatment^28^. To gain a more in-depth understanding of barriers to treatment access and their underlying reasons, open-ended questions adapted from a UK study on healthcare experiences in Long COVID were included in the survey. These items complemented the structured data by allowing participants to report and elaborate on perceived barriers to care^28^.

The Illness Invalidation Inventory (3*I) was used to assess MI from various social sources^22^. The 3*I is validated in different languages, including German^29^. Eight items were collected for each source (medical staff, family, partner, government agencies, and work environment). These eight items per social source can be divided into two subscales: Five items form the Discounting subscale, which reflects the active downplaying or devaluation of the illness by the respective source. The remaining three items belong to the Lack of Understanding subscale and capture a perceived lack of empathy or understanding. As the instrument already is validated, validating measures (factor analysis, convergent validity using correlations) are reported in Appendix 9–10.

#### Experienced social support

To measure online community use, we used questions from the “Health Information National Trends Survey (HINTS) Germany”^30^, developed by the Foundation for Health Knowledge, specifically from the “Use of Social Networks” category.

### Response rate and case selection

A total of 908 individuals subscribed to the survey. 38 participants closed the survey immediately after accessing the first page and did not provide any content. After applying the inclusion and exclusion criteria and excluding incomplete questionnaires and duplicates, we obtained a final sample of 577 participants to be analyzed (Appendix 3). The following analyses and results refer exclusively to the final sample.

### Data analysis

Statistical analyses were performed using IBM SPSS Statistics Version 31.0.0.0. First, descriptive statistics were calculated for all variables. To examine group differences between the diagnostic groups (LC/PC, ME/CFS, and PACVS), one-way analyses of variance were performed. In cases of violation of variance homogeneity, the robust Welch ANOVA was used, followed by Games-Howell post hoc tests. For categorical variables, group differences were examined using chi-square tests (χ²) and Cramér’s V was calculated as the effect size. For ordinal or non-normally distributed metric variables, Mann-Whitney U tests (for two groups) or Kruskal-Wallis tests (for three or more groups) were used.

Correlations between variables were examined using correlation analyses. Spearman rank correlations were calculated for correlations between ordinal scaled variables. Pearson correlations were calculated when dichotomous variables were correlated with metric variables. The significance level was set at *p* < 0.05. For multiple statistical tests, a Bonferroni correction was applied to adjust the significance level. Open-ended free-text responses were evaluated using conventional qualitative content analysis^31^. The responses were first read in their entirety and then assigned to categories. The categories were formed in such a way that all responses from participants that differed in content could be represented. The categories were then summarized and evaluated descriptively.

## Results

### Demographic characteristics

The sociodemographic characteristics of the participants are shown in Table 1. The participants were spread across all age groups, with the majority aged between 40 and 65. The sample included participants of all genders. Around 83% were female. The sample represented various levels of education, including school, vocational, and academic qualifications. The participants represented various marital statuses. Just under half of the participants stated that they had children. 94.2% of respondents reported that they had been employed or in training or studying before the illness, while only 31.1% reported that this was the case after the illness. Changes in employment status are shown in Table 1 and visually illustrated in Appendix 4.

Most of the participants (85.1%) reported confirmed diagnoses of PASC and PACVS and 53.4% of respondents stated that they did not notice any change in their health status after treatment, whereas 37.3% stated that their condition had deteriorated.

**Table 1:** Sociodemographic and medical characteristics of *N* = 577 individuals affected by PAIMS.

Characteristic		*n* (%)	
Total		577 (100)	
Gender Female		482 (83.5)	
Male		90 (15.6)	
diverse		5 (0.9)	
Age groups 18–28		42 (7.3)	
29–39		108 (18.7)	
40–50		157 (27.2)	
51–65		252 (43.7)	
> 65		18 (3.1)	
Marital status			
Single		168 (29.1)	
Married / Cohabiting / Partnership		344 (59.6)	
Separated/divorced		59 (10.2)	
Widowed		6 (1)	
children Total		303 (52.5)	
1 child		162 (53.4)	
2 children		87 (28.7)	
≥ 3 children		54 (17.8)	
Education			
No school leaving certificate		1 (0.1)	
Secondary school or secondary school diploma		82 (14.2)	
(Technical) high school diploma		94 (16.3)	
Vocational training		128 (22)	
University degree		244 (42.3)	
Doctorate		28 (4.9)	
Occupation before/after diagnosis		Before	After
Full-time employee		281 (48.8)	50 (8.7)
Part-time employees		176 (30.5)	91 (15.8)
Self-employed		41 (7.1)	18 (3.1)
Unemployed/job seeker		6 (1)	52 (9)
In higher education/training		48 (8.3)	20 (3.5)
Retired		19 (3.3)	214 (37.1)
Housewife/househusband		6 (1)	10 (1.7)
Other (e.g., unable to work)		n/a	122 (21)
Severity of symptoms of last COVID infection*			
Hardly any symptoms		7 (1.2)	
Mild		238 (41.2)	
Moderate		195 (33.8)	
Severe		75 (13)	
Very severe		13 (2.3)	
Change in condition after treatment*			
Improvement		128 (22.2)	
Deterioration		215 (37.3)	
No change		308 (53.4)	
Diagnosis related to COVID (vaccination)*			
LC/PC	Confirmed	192 (33.3)	
	Suspected	27 (4.7)	
ME/CFS	Confirmed	256 (44.3)	
	Suspected	37 (6.4)	
PACVS	Confirmed	43 (7.5)	
	Suspected	22 (3.8)	

* Information on the symptoms of the last COVID-19 infection, the change in condition after treatment, and whether the disease was suspected or had already been confirmed was based on participant self-report.

### Healthcare access, utilization, and care experiences

#### Diagnostic structures and specialties

LP/PC and ME/CFS were most frequently diagnosed by general practitioners (40% / 37%) or neurologists (24% / 28%), while PACVS was most frequently diagnosed by neurologists, general practitioners and cardiologists (33% / 20% / 30%). Only a minority of the participants (12% each of LC/PC and ME/CFS, 7% of PACVS) were diagnosed in syndrome-specialised outpatient clinics (Table 2). Other specialties are shown in Table 2 and appendix 5 illustrates the findings.

**Table 2:** Visited diagnostic specialist practices and outpatient clinics among individuals affected by LC/PC, ME/CFS, and PACVS.

Specialty	LC/PC %	ME/CFS %	PACVS %	X² (*p*)
Post-COVID outpatient clinic	12.1	12.2	2	
Post-vaccination clinic	n/a	n/a	4.7	
General practitioner	40	37.4	30.2	5.06 (0.79)
Neurology	24.2	27.6	32.6	0.63 (0.73)
Internal medicine	14.2	16.5	9.3	3.2 (0.196)
Pulmonology	7.4	5.9	7	2.3 (0.303)
Rehabilitation facility	7.4	3.9	0	5.9 (0.51)
Immunology	1.6	4.7	11.6	6.6 (0.035)*
Angiology	0	2.8	7	
Infectious diseases	2.1	1.6	7	
Cardiology	5.8	3.1	30.2	30.4 (< 0.001)***
Psychiatry	6.3	5.1	7	0.3 (0.85)
Rheumatology	0.5	0.4	4.7	

Note: Of a total of 47 (Appendix 6) medical specialties, outpatient clinics, and rehabilitation facilities that were visited by those affected for diagnosis, the most frequently mentioned are listed in this table. Participants were able to list several facilities that they consulted during the course of their illness. Overall, PACVS sufferers reported having visited more different specialties than those affected by LC/PC or ME/CFS. The pairwise comparisons between the diagnostic groups regarding visits to cardiologists and immunologists are presented in appendix 7. Empty fields: Due to low expected cell frequencies, the analyses were limited to dichotomous variables (“subject area attended: yes/no”). * *p* ≤ 0.05, ** *p* ≤ 0.01, *** *p* ≤ 0.001.

#### Treatment and service utilization

The use of general practitioner and specialist care was high in all diagnosis groups (LC/PC: 91.7%; ME/CFS: 95.1%; PACVS: 93.7%). In addition, specialized outpatient clinics were used by 38.5% of LC/PC and 35.2% of ME/CFS patients, as well as by 50.8% of PACVS patients. There were significant differences between PACVS and ME/CFS (χ² = 7.3; *p* = 0.007; Cramér’s V = 0.14) and between PACVS and LC/PC (χ² = 4.7; *p* = 0.029; Cramér’s V = 0.13).

Therapeutic interventions targeting the musculoskeletal system (including occupational therapy, physiotherapy, and chiropractic care) were utilized by 4.4% of individuals with LC/PC and 6.0% of those with ME/CFS, but by none of the PACVS group. The use of psychotherapeutic services were reported by 14.6% of LC/PC, 10.5% of ME/CFS, and 9.5% of PACVS participants. Across all diagnostic groups, the use of complementary and alternative medicine was low, ranging from 1% to 5%.

#### Barriers to therapeutic care

87% of all participants stated that they had experienced difficulties in obtaining the therapy they needed (LC/PC 80.8%, ME/CFS 91.1%, PACVS 92.3%, x² 13.6, p 0.001 Cramer-V 0.154). There is a significant difference between LC/PC and PACVS sufferers (x² 4.7, p 0.029) and between LC/PC and ME/CFS sufferers (X² 11.5 *p* < 0.001, Cramer-V 0.15). An overview of the reasons given can be found in Table 3.

**Table 3:** Reported difficulties in obtaining the necessary therapy among individuals affected by PAIMS.

Reasons	Frequency *
No treatment options available	60% (*n* = 346)
Own symptoms were not considered serious enough	42.6% (*n* = 246)
Did not know who to contact	36.4% (*n* = 210)
The doctor does not have enough budget for therapy	15.3% (*n* = 88)
Refusal of treatment due to the illness	14% (*n* = 81)
Feeling of not being sick enough	8.8% (*n* = 51)

*Frequencies are presented as percentages and absolute numbers (n) of participants reporting each reason. Multiple responses were possible.

Other reported barriers primarily concerned structural obstacles to accessing care. Overall, 87.9% of participants across all diagnostic groups experienced such barriers, with a significant difference between groups (χ² = 5.5, *p* = 0.018, Cramér’s V = 0.14). These were reported by 84.0% (*n* = 184) of LC/PC, 89.1% (*n* = 261) of ME/CFS, and 95.4% (*n* = 62) of PACVS participants (Table 4).

A key barrier was the reluctance of healthcare professionals to engage with these conditions, which differed significantly between groups (χ² = 9.8, *p* = 0.007), particularly between ME/CFS and LC/PC as well as ME/CFS and PACVS. This was reported by 33.1% of ME/CFS participants.

Travel and accessibility difficulties were also more frequently reported by ME/CFS participants compared to LC/PC and PACVS, with a significant difference between ME/CFS and LC/PC (χ² = 6.1, *p* = 0.013).

Based on responses to an open-ended question, 26.2% of participants across all groups reported feeling not being taken seriously during medical consultations. 12.1% indicated that their symptoms were prematurely attributed to psychological causes, and 20.3% reported insufficient health insurance coverage as a barrier.

A further barrier was the perceived knowledge of the physician about the disease. This knowledge was perceived low by participants. (Likert scale 1 = satisfied to 5 = not satisfied; LC/PC: M = 3.93; ME/CFS: M = 3.92; PACVS: M = 4.42), with no significant difference between groups (H = 4.9; *p* = 0.19).

**Table 4:** Structural barriers to accessing treatment among individuals affected by LC/PC, ME/CFS, and PACVS assessed by open-ended questions.

Barrier	LC/PC % (*n*)	ME/CFS % (*n*)	PACVS % (*n*)	X² (*p*)
Health insurance does not cover the costs	18.3 (40)	21.2 (62)	23.1 (15)	1.0 (0.6)
No available healthcare provider or therapeutic options	17.8 (39)	17.1 (50)	24.6 (16)	2.0 (0.35)
Doctors do not pursue further training	21.9 (48)	33.1 (97)	20 (13)	9.8 (0.007)**
Not being taken seriously	24.2 (53)	25.9 (76)	33.8 (22)	2.4 (0.3)
Barriers and access to healthcare facility	4.6 (10)	10.6 (31)	3.1 (2)	8.6 (0.014)*
No available dates	16.9 (37)	14.7 (43)	13.8 (9)	0.6 (0.73)
Symptoms labeled as psychological	10.5 (23)	11.9 (35)	18.5 (12)	2.9 (0.22)
No prescription of medication	5 (11)	15 (15)	0	3.5 (0.17)

Note: This table presents responses to an open-ended questionnaire item. Values are presented as percentages and absolute numbers (n). Differences between diagnostic groups were assessed using chi-square tests. * *p* ≤ 0,05, ** *p* ≤ 0,01, *** *p* ≤ 0,001.

### Experiences with medical invalidation

#### Perceived consideration of symptoms in treatment

Overall, about a quarter of respondents reported feeling that their symptoms were taken seriously (LC/PC: 25.6%; ME/CFS: 29.0%; PACVS: 18.4%), while about half reported feeling that their symptoms were hardly taken seriously or not taken seriously at all (LC/PC: 50.2%; ME/CFS: 48.1%; PACVS: 46.1%). There was no significant difference between the diagnostic groups (H (2) = 1.38, *p* = 0.502). In addition, participants in all three diagnostic groups reported that their symptoms were downplayed or attributed to psychological factors (e.g., anxiety) (LC/PC: 82.2%, *n* = 180; ME/CFS: 86.7%, *n* = 254; PACVS: 93.8%, *n* = 61). The group difference was not statistically significant (χ² = 6.4; *p* = 0.172).

When asked whether all the participants’ symptoms were considered during treatment, the majority of the three diagnostic groups responded that this was not the case (LC/PC 59.7% (*n* = 85), ME/CFS 55.5% (*n* = 157), PACVS 60% (*n* = 39)). There were no significant group differences and a very small effect, X² ,06, *p* = 0.588, Cramer V 0.044. Respondents in all three diagnostic groups reported that they did not have a specific treatment plan during their treatment (LC/PC 37.5%, ME/CFS 52.6%, PACVS 50%).

When looking at the symptoms reported by those affected that were not considered in the treatment plan, there was no significant difference between the diagnostic groups (Table 5).

Participants in all three diagnostic groups (LC 6.7%, ME/CFS 4.6%, PACVS 7.9%) describe that they only received symptomatic therapy, but no therapy for the underlying disease. Similarly, participants (LC 4.2%, ME/CFS 3.9%, PACVS 10.5%) found that they had difficulty obtaining holistic treatment. Thus, doctors only looked at organs within their own area of expertise without considering complaints regarding other organs.

**Table 5:** Reported symptoms that participants perceived as not being adequately considered during medical treatment.

	LC/PC	ME/CFS	PACVS	Chi² (*p*=)
Fatigue/weakness	22.5% (*n* = 27)	15.8% (*n* = 24)	10.5% (*n* = 4)	3.45 (0.18)
PEM	14.2% (*n* = 17)	14.5% (*n* = 22)	10.5% (*n* = 4)	0.91 (0.9)
Pain	10% (*n* = 12)	9.9% (*n* = 15)	2.6% (*n* = 1)	1.77 (0.41)
Muscular/ orthopedic symptoms	12.5% (*n* = 15)	5.9% (*n* = 9)	10.5% (*n* = 4)	4.14 (0.12)
Postural tachycardia syndrome (POTS)	1.7% (*n* = 2)	7.9 (*n* = 12)	2.6% (*n* = 1)	

Note: Reported symptoms that participants perceived as not being adequately considered during medical treatment among individuals with LC/PC, ME/CFS, and PACVS. Data are presented as percentages and absolute numbers (n). Differences between groups were examined using chi-square tests. Empty fields: Cell (25.0%) expected frequency < 5, Chi² condition violated. * *p* ≤ 0,05, ** *p* ≤ 0,01, *** *p* ≤ 0,001.

#### Perceived invalidation of illness by source

Perceived invalidation by medical personnel was moderate in all diagnostic groups (LC: M = 2.80 ± 0.94; ME/CFS: M = 2.99 ± 1.02; PACVS: M = 3.03 ± 0.84) and did not differ significantly between the diagnostic groups (Welch-F (2, 190.50) = 2.87, *p* = 0.059). Descriptively, ME/CFS and PACVS patients showed higher values than LC patients.

Perceived invalidation by authorities (e.g. disability benefit agencies or welfare & social work programs) differed significantly between diagnostic groups (Welch-F = 5.19, *p* = 0.007) (Table 6). Post-hoc analyses (Games-Howell) revealed that ME/CFS sufferers reported higher invalidation than LC/PC sufferers (*p* = 0.006), while PACVS sufferers did not differ significantly from the other two groups.

**Table 6:** Mean illness invalidation (3*I) scores by source and diagnostic group.

Source	LC/PC M ± SD	ME/CFS M ± SD	PACVS M ± SD	Welch-F (*p*)
Partner	1.96 ± 0.88	2.06 ± 0.95	2.02 ± 0.76	0.56 (0.57)
Family	2.64 ± 1.0	2.54 ± 1.0	2.38 ± 0.85	1.97 (0.14)
Medical staff	2.8 ± 0.94	2.98 ± 1.01	3.03 ± 0.83	2.86 (0.059)
Work	3.26 ± 1.05	3.27 ± 0.95	3.21 ± 0.95	0.44 (0.95)
Authorities	3.22 ± 1.01	3.58 ± 1.0	3.58 ± 0.83	5.2 (0.007)**

Note: Mean levels of illness invalidation (3*I) reported by participants across different sources (partner, family, medical staff, workplace, and authorities) and diagnostic groups (LC/PC, ME/CFS, and PACVS). Data are presented as mean ± (likert-scala 1 never − 5 often) standard deviation (M ± SD). the higher the mean, the higher the invalidation. Differences between groups were tested using Welch’s ANOVA. * *p* ≤ 0,05, ** *p* ≤ 0,01, *** *p* ≤ 0,001.

When considering invalidation independently of the diagnostic group to determine which social category exerts the most medical invalidation on those affected, significant differences between the sources of invalidation were found (χ² = 172.03, *p* < 0.001; Friedman test). The highest mean ranks (range: 1–5) were found for authorities (MR = 3.73) and the work environment (MR = 3.67), followed by medical staff (MR = 3.23), while family (MR = 2.70) and especially partners (MR = 1.67) had the lowest scores.

The detailed results of the subscales of the 3*I (Discounting and Lack of Understanding), including group comparisons, are listed in appendix 8.

### Role of online community use

The use of social networks such as Facebook and LinkedIn to seek self-help differed significantly between the three diagnostic groups, χ² (2) = 12.06, *p* = 0.002, Cramér’s V = 0.15. ME/CFS sufferers reported the most frequent use (89.4%), followed by PACVS (82.3%) and LC/PC (78.2%). The difference in usage between ME/CFS and LC/PC sufferers proved to be significant (X² 11.9, p = < 0.001) with a small effect size (Cramer’s V 0.153). (Table 7)

**Table 7:** Reported use of social media and online platforms among individuals affected by LC/PC, ME/CFS, and PACVS.

Question	LC/PC (*n*)	ME/CFS (*n*)	PACVS (*n*)	Chi² (*p*=)	Cramer V
Facebook/LinkedIn visited	78.2 (169)	89.4 (262)	82.3% (51)	12.05 (0.002)**	0.145 (0.002)**
Sharing health information with others	67.1% (145)	74.7% (219)	75.8% (47)	4.08 (0.13)	0.085 (0.13)
Writing an online diary/blog	10.2% (22)	13.7% (40)	17.7% (11)	2.87 (0.238)	0.071 (0.238)
Online forum/self-help group to obtain information and exchange ideas	90.3% (195)	90.8% (266)	88.7% (55)	0.25 (0.88)	0.02 (0.88)
Watch YouTube video	79.6% (172)	83.3% (244)	85.5% (53)	1.66 (0.43)	0.054 (0.43)

Note: Reported use of social media and online platforms for information exchange among individuals with LC/PC, ME/CFS, and PACVS. Data are presented as percentages and absolute numbers (n). Differences between groups were examined using chi-square tests, with Cramer’s V reported as a measure of effect size. * *p* ≤ 0,05, ** *p* ≤ 0,01, *** *p* ≤ 0,001.

## Discussion

The results of this study show that individuals with LC/PC, ME/CFS after COVID-19 infection, and PACVS in Germany face a variety of challenges in their medical care. Many participants reported difficulties in accessing appropriate treatment, including problems with scheduling appointments, perceived insufficient knowledge of the conditions on the part of medical staff, and experiences of their symptoms being dismissed, downplayed, or psychologized. These experiences were accompanied by a perceived MI by treating staff. In addition, invalidation was also reported in other social contexts, such as in the workplace or when dealing with authorities. Concurrently, many participants reported an increased reliance on peer support, particularly through social media platforms.

Our findings identify not being taken seriously and medical invalidation (MI) as key structural barriers in the care of PAIMS conditions. Across data sources, reports of MI were consistently high, indicating that affected individuals are frequently required to legitimize their symptoms within healthcare and institutional contexts. These findings align with previous qualitative research in ME/CFS, which documents recurrent forms of invalidation, including premature psychologization, patient-blaming, and insufficient clinical knowledge^14,21^. While the assessment of psychological symptoms constitutes an essential component of comprehensive clinical care, premature attribution of symptoms to psychological causes—without careful consideration of underlying neuropsychiatric pathology and differentiation between PAIMS-related manifestations and comorbid conditions—requires particular caution^32^. A large quantitative cross-sectional study conducted in Germany also identified downplaying and psychologization as a highly relevant phenomena within LC/PC affected individuals after the pandemic^19^. Similar patterns of invalidation have also been observed in other post-infectious conditions, such as Lyme disease^33^. Collectively, this evidence underscores that delegitimization and lack of recognition constitute a pervasive and systemic challenge in the care of complex post-infectious diseases.

The results indicate that experiences of invalidation extend beyond medical settings to broader social and institutional contexts. Notably, participants reported comparatively higher levels of invalidation in interactions with authorities than with medical staff, partners, family, or the workplace. This pattern is broadly consistent with earlier research using the Illness Invalidation Inventory (3*I). In that study, patients with rheumatoid arthritis and fibromyalgia most frequently reported invalidation from social institutions, workplaces, and family members, whereas HCPs were less frequently identified as a source of invalidation^22^. These findings underscore the significant role of institutional contexts in shaping experiences of invalidation. However, the relative importance of different sources of invalidation differs from prior findings. While medical personnel played a minor role in earlier research^22^, they emerged in the present study as a major source of invalidation, second only to authorities and the work environment. In contrast, family members and partners were least frequently identified as sources of invalidation among individuals with PAIMS. This pattern highlights the particularly prominent role of institutional and healthcare contexts in shaping invalidation experiences. A possible explanation is the limited institutional recognition and experience with these conditions, particularly PACVS^34^. Similarly, individuals with ME/CFS frequently report a lack of recognition in administrative and social systems, potentially due to the often invisible nature of symptoms such as fatigue and cognitive impairment^35^. These factors may increase the need for patients to repeatedly justify their illness in institutional and occupational settings, thereby contributing to heightened emotional burden and reduced well-being.

These findings are further reflected in the work context. Participants reported substantial invalidation in the workplace, and many who had been employed prior to illness onset reduced their working hours or left the workforce altogether. A considerable proportion also reported ongoing inability to work, with some describing exclusion from social security systems and delays in administrative processes. These results are consistent with previous research, particularly in ME/CFS, where high rates of unemployment due to illness-related limitations have been documented^36^. Accordingly, studies highlight the need for workplace adaptations, improved employer support, and greater awareness to facilitate sustained occupational participation^23,37^.

Our findings reveal a discrepancy between open-ended responses and direct questioning regarding experiences of MI. While approximately one quarter of participants spontaneously reported not being taken seriously or having their symptoms psychologized, a substantially larger proportion confirmed such experiences when asked explicitly. This suggests that affected individuals may not readily articulate these experiences unless prompted and may instead frame them in terms of broader structural deficiencies within the healthcare system. These include limited disease knowledge, administrative barriers, and restricted access to care^38^, which can delay or prevent treatment and exacerbate health and work impairments. Similar patterns have been reported in studies on LC/PC, where patients face skepticism, limited clinical understanding, and a lack of clear treatment pathways^4^. Despite increased awareness since the early pandemic phase, our results indicate only modest improvements in care: approximately one-third of participants reported lacking both a clear treatment plan and an appropriate point of contact. Overall, these findings highlight persistent structural challenges in the management of PAIMS, underscoring the need for expanded specialized care.

Many affected individuals use social media platforms (e.g., Facebook or LinkedIn) to exchange information on perceived shortcomings in healthcare and institutional systems. Within these online communities, they share experiences with healthcare providers, identify specialized services, and provide guidance on administrative processes and symptom management. Consistent with previous research, which conceptualizes such groups as patient-led epistemic communities^21^, participants in the present study also reported high levels of engagement with these platforms. These communities thus serve as important spaces for exchanging experiential knowledge, particularly in the context of medical invalidation and limited formal care.

This study has several notable strengths. It provides a comparative analysis of PACS and PAVCS in the context of the COVID-19 pandemic, examining differences in care experiences across post-infectious and post-vaccination conditions. With a relatively large sample (*N* = 577), it captures a broad range of patient experiences. By combining standardized measures (e.g., the 3*I) with open-ended responses, the study integrates quantitative findings with insights into individual perspectives. It also highlights structural challenges within the health and social care system, an area that remains underexplored in existing research.

However, several limitations should be considered when interpreting the results. Due to the cross-sectional study design, no causal relationships between invalidation experiences and health or social consequences can be inferred. In addition, the data collected is based on self-reports from participants, which means that subjective perceptions or memory effects could have influenced the results^39^. In addition, diagnoses of Long COVID and Post COVID were self-reported and were not independently verified. Given the inconsistent application of these terms in routine clinical practice, some diagnostic misclassification between these groups cannot be excluded.

Recruitment via online platforms may also have led to selection bias^40^, as individuals with particularly pronounced experiences or stronger connections to patient communities may have been more likely to participate in the study. In addition, recruitment through online patient communities may have contributed to the high proportion of female participants, although this cannot be determined from the present study. Furthermore, the diagnostic groups examined varied in size (with the group of PACVS sufferers being the smallest), which may limit the comparability of the results^41^. Furthermore, this study included adults only. Qualitative evidence suggests that children and adolescents with postacute immune mediated syndromes may experience similar or even greater challenges related to healthcare access^42^, highlighting the need for future quantitative research in pediatric populations. Finally, the umbrella term PAIMS is used here as a proposed conceptual framework to facilitate discussion of the shared healthcare experiences of clinically related postacute conditions associated with the COVID-19 pandemic. It is intended solely for the purposes of this study and should not be interpreted as an established diagnostic classification, as encompassing all postinfectious syndromes, or as evidence of a shared pathophysiological mechanism.

These findings have important implications for medical care, policy and research. Participants consistently expressed the need for more intensive and continuous training of medical staff to improve the management of these conditions, in line with previous research. This need may partly reflect the limited coverage of postviral conditions such as ME/CFS in undergraduate medical education, where these conditions have historically received little attention and, when taught, have often been addressed inconsistently across different medical specialties^43^. Participants in the present study also emphasized the importance of clear, comprehensible communication and well-structured treatment plans. Although recommendations and guidelines^44–47^ exist for some of these conditions, both our findings and recent studies^48,49^ suggest that they are not yet sufficiently disseminated or consistently implemented in routine clinical practice.

The findings indicate structural barriers within the health and social care system that cannot be addressed at the individual level and require policy-level interventions. There is a clear need to expand specialized care structures, particularly outpatient services that provide both diagnosis and ongoing management for individuals with PASC and PACVS. Furthermore, formal institutional recognition of these conditions is essential to improve access to social security benefits and to prevent administrative disadvantages arising from unclear responsibilities and limited clinical awareness.

Previous research indicates that experiences of MI have not only psychosocial but also physiological effects that may contribute to symptom exacerbation^50^. For example, studies on chronic pain conditions such as fibromyalgia demonstrate that physical and social pain are closely interconnected and share partially overlapping neural mechanisms^51^. Given the high prevalence of invalidation observed in the present study, there is a clear need for longitudinal research in PASC and PACVS populations to examine its long-term impact. In particular, such studies should investigate how repeated invalidation influences disease progression, symptom severity, and psychosocial burden. In parallel, further research into underlying pathomechanisms is essential to enable the development of targeted treatments that extend beyond symptomatic management toward causal therapeutic approaches.

## Conclusion

The findings provide clear evidence of structural deficiencies in the care of PAIMS conditions. Affected individuals frequently report not being taken seriously and experiencing MI, highlighting these as central challenges within healthcare provision. These gaps are partially mitigated by reliance on social and peer support networks. In particular, insufficient clinical knowledge and institutional barriers likely contribute to the inadequate recognition and misclassification of symptoms.

## Supplementary Information

Below is the link to the electronic supplementary material.

Supplementary Material 1

Supplementary Material 2

## Data Availability

The data is available with restricted access. The surveys that support the findings of this study are available from the last author of the study, but restrictions apply due to the ethics committee decision and the decision of the data protection officer. The data are only available upon reasonable request from the last author of the study.
