# Supplementary material for: Medical invalidation is associated with structural barriers in postacute immune mediated syndromes based on patient perspectives in Germany
Source: Sci Rep. 2026 Aug 25;16:26687. doi: 10.1038/s41598-026-67883-2 (PMC13507045; doi:10.1038/s41598-026-67883-2)
Supplement: Supplementary file 1 [file 41598_2026_67883_MOESM1_ESM.docx]

Appendix 1 of the Manuscript “Medical invalidation is associated with structural barriers in postacute immune mediated syndromes based on patient perspectives in Germany”

**Study Questionnaire: Experiences of People with Long-/Post-COVID, ME/CFS and post-acute COVID-19 vaccination syndrome in the Healthcare System in Germany: Care, Communication, and Relationships with Doctors**

**Note:** The original survey was administered in German. The version provided here is an English translation for publication purposes only. Questions included in the present study and analyses are highlighted in yellow.

1. ***Category: Demographics***

**Your gender:**

- Female
- Male
- Diverse

**Your age:**

- 18–28 years
- 29–39 years
- 40–50 years
- 51–65 years
- Older than 65

**Your marital status:**

- Single
- Married **/** Cohabiting / partnership
- Separated **/** Divorced
- Widowed

**Do you have children?**

- Yes
- No

**If yes, how many:______**

**Your highest level of education:**

- No school leaving certificate
- Secondary school or secondary school diploma
- (Technical) high school diploma
- Vocational training
- University degree
- Doctorate

**Your employment status before Long/Post-COVID, ME/CFS, or vaccine-related injury:**

- Full-time employee
- Part-time employee
- Self-employed
- Unemployed/job seeker
- In higher education/training
- Retired
- Housewife/househusband

If employed part-time, how many hours per week did you work previously?___

**Your employment status following a diagnosis of Long/Post-COVID, ME/CFS, or vaccine injury (your current employment status):**

- Full-time employee
- Part-time employee
- Self-employed
- Unemployed/Job seeker
- In higher education/training
- Retired
- Housewife/househusband
- Other______

If employed part-time, how many hours do you currently work per week?___

___________________________________________________________________

***Category 2: Physical and mental well-being in connection with Long/Post-COVID, ME/CFS, or vaccine-related injuries***

1. Have you ever tested positive for COVID-19?
   - Yes
   - No
2. If yes, how long ago was that (the last time)? _____
3. If yes, how many times have you been infected with COVID-19?____
4. If yes, what was the course or severity of your most recent COVID-19 illness?____
5. Which of the following diagnoses currently apply to you?

- Post-COVID/Long COVID (confirmed)
- ME/CFS (confirmed)
- Post-COVID/Long COVID (suspected)
- ME/CFS (suspected)
- Medically diagnosed vaccine-related injuries in cases of persistent symptoms following a COVID-19 vaccination (e.g., post-vaccination syndrome)
- Clinically suspected vaccine-related adverse events with persistent symptoms following a COVID-19 vaccination
- If a diagnosis of Post-COVID or Long COVID or ME/CFS has been made: How long after the onset of the first symptoms was the diagnosis made?____
- If there is a medically diagnosed case of vaccine-related injury: How long after the onset of the first symptoms was the diagnosis made?_____
- **Other -> End of the survey**

6. If any of these diagnoses applies, when was the diagnosis made? ______

7. What was the medical specialty of the doctor who made your current diagnosis related to Long/Post-COVID or the vaccination? Please list only the specialty here; do not include names

8. Before your current diagnosis, were any other diagnoses related to your COVID-19 illness made that are no longer relevant?

- Yes
- No

If yes, when?___

If yes, by whom? ____

If yes, what additional diagnoses were made that are no longer current?

- ME/CFS
- Post-COVID/Long COVID
- Post-vaccination syndrome
- Encephalitis
- Other:______

9. Have you been diagnosed with any other conditions since your COVID-19 infection that are not directly related to COVID-19?

- Yes
- No

If yes, which ones? ____

If yes, by whom (which specialist)? ___

10. Did you have any medically diagnosed illnesses or conditions prior to your COVID infection or vaccine-related adverse events? If yes, which of the following apply? (Multiple answers allowed)

- Cardiovascular diseases (for example: high blood pressure, arrhythmia, heart failure, stroke / TIA)
- Respiratory diseases (e.g., asthma, COPD, chronic bronchitis)
- Metabolic / hormonal disorders (e.g., diabetes, thyroid disorders)
- Autoimmune diseases (e.g., rheumatoid arthritis, multiple sclerosis, inflammatory bowel disease, systemic lupus erythematosus)
- Mental health conditions (for example: depression, anxiety disorders, PTSD, burnout)
- Obesity
- Smoker
- No pre-existing conditions
- Other:____

***3. Health Issues and Treatment***

11. Health problems caused by Long/Post-COVID-19, ME/CFS, or vaccine-related injuries.

The health issues listed below refer to limitations caused by your condition that you have noticed. Please indicate how much of a problem these have been for you over the past 12 months. No problem (1) to extreme problem (5)

- Sleep disturbances
- Limited mobility
- Muscle problems
- Respiratory problems
- Impaired sense of smell and/or taste
- Hair loss
- Tinnitus
- Dizziness
- Heart palpitations
- Pain
- Fatigue
- Difficulty concentrating
- Mood swings
- Lack of motivation
- Anxiety/worries
- Fatigue
- Post-exertional malaise

12. In which areas of the body are you currently experiencing pain? (Multiple answers allowed)

- Neck/Shoulder
- Lower back
- Upper back
- Joints
- Head
- Abdomen
- Whole body
- Other
- None

13. In which areas of the body have you experienced pain in the past? (Multiple answers allowed)

- Neck/Shoulder
- Lower back
- Upper back
- Joints
- Head
- Abdominal Poses
- Whole body
- Other
- None

14. On average, how severe was your pain over the past 5 days?

- Scale 1 to 10

15. Did you have difficulty, in your opinion, obtaining necessary therapeutic measures, assistive devices, and/or medical treatment?

- No
- Yes.

16. For what reasons did you not receive the treatment you needed? (Multiple answers allowed)

- I was denied treatment due to my COVID-19 illness/post-vaccination syndrome.
- I didn’t know who to contact.
- There were no treatment options available
- I was told that the doctor didn’t have enough budget for therapy.
- I thought I wasn’t sick enough.
- My symptoms were not considered severe enough.
- Other reasons (free-text field)

17. Which of the following statements apply to your treatment so far? (Multiple selections allowed)

- The therapy was rather exhausting.
- The rest breaks I needed due to fatigue were not taken seriously.
- The treatment did not lead to any improvement—my condition remained the same
- The treatment led to a worsening of my condition.
- The treatment led to an improvement in my condition.

18. How much have the following symptoms affected you over the past 2 weeks? Scale 0 (Not at all) to 3 (Almost every day)

- Little interest or pleasure in doing things
- Feeling down, depressed, or hopeless
- Feeling nervous, anxious, or on edge
- Not being able to stop or control worrying

19. For each of the following statements, please check how often you have felt this way over the past two weeks. Use the following scale: never: 1 = never, 2 = almost never, 3 = sometimes, 4 = quite often, 5 = very often.

- In the last month, how often have you felt that you were unable to control the important things in in your life
- In the last month, how often have you felt confident about your ability to handle your personal problems?
- In the last month, how often have you felt that things were going your way?
- In the last month, how often have you been angered because of things that happened that were outside of your control?

20. For each of the following statements, please check how often you have felt this way over the past two weeks. Use the following scale 1 = not at all true, 2 = not very true, 3 = some true, 4 = somewhat true, 5 = completely true

- I suddenly have intrusive thoughts or memories that cause me significant distress.
- I avoid certain places, situations, or conversations without knowing exactly why.
- I am often overly alert or easily startled, even in situations that are actually safe.
- I feel “numb” or emotionally detached.

________________________________________________________________________________

***Category 4: The Healthcare Situation from Your Perspective***

1. Have you sought medical help due to Long/Post-COVID or ME/CFS symptoms, or due to medically confirmed vaccine-related injuries?
   - No
   - Yes
2. Have you sought other forms of help due to Long/Post-COVID or ME/CFS symptoms, or due to medically confirmed vaccine-related injuries?
   - No
   - Yes, the following: ____________________________________________
3. Is your treatment currently being supervised by a doctor?
   - Yes
   - No
4. Were all of your symptoms taken into account when your treatment plan was developed?
   - Yes
   - No

If no, which ones were not? _____

5. Have you experienced any obstacles to receiving care for your long/post-COVID, ME/CFS, or vaccine-related health issues?
   - Yes
   - No

If yes, which ones?_____

6. Have you experienced any medical gaslighting i.e. where someone has blamed your symptoms on psychological factors such as anxiety?
   - Yes
   - No
   - I would prefer not to answer this question
7. Please fill in or check the answer that applies to you.

Scale: Agree (1) – 2 – 3 – 4 – Disagree (5)

- I feel that my symptoms are taken seriously.
- My views are taken into account in decisions regarding my further medical care.
- I am encouraged to actively participate in my treatment plan
- I am satisfied with the scope of the examinations performed.
- I am satisfied with the initiation of therapeutic measures (e.g., respiratory therapy, physical therapy, rehabilitation).
- I am made aware of self-help resources.
- My primary care physician takes on the responsibility of organizing my overall treatment
- I am satisfied with the exchange of information among the treating physicians.
- I am satisfied with the level of expertise of the treating physicians.

8. Is there anything else you would like to tell us about your treatment that has not been discussed so far? _______

9. We are interested in how others react to people with health problems or an illness. The following sections refer to various people in your life. Please rate how often these individuals or groups of people have reacted to you in the manner described over the past year.

After each statement, circle a number from 1 (never) to 5 (very often) to indicate how often this reaction occurred.

The questionnaire consists of five sections. You will rate the same reactions multiple times, each time with regard to different people. If a particular section does not apply to you, you may skip that part and continue with the next section. Remember: Rate the statements based on how others have reacted to you as a person with health problems or an illness.

**Section 1**: Spouse or Partner

If you are single (not married, widowed, or in a committed relationship), please skip Section 1 and go directly to Section 2.

My spouse or partner……

- finds it odd that I can do much more on some days than on other days
- thinks I should be tougher.
- takes me seriously.
- gives me unhelpful advice.
- understands the consequences of my health problems or illness.
- makes me feel like I’m exaggerator.
- thinks I can work more than I do.
- gives me the chance to talk about what is on my mind.

**Section 2:** Family, for example, children, parents, siblings, uncles, aunts, grandparents, in-laws.

 My family……

- finds it odd that I can do much more on some days than on other days
- thinks I should be tougher.
- takes me seriously.
- gives me unhelpful advice.
- understands the consequences of my health problems or illness.
- makes me feel like I’m exaggerator.
- thinks I can work more than I do.
- gives me the chance to talk about what is on my mind.

**Section 3**: Healthcare professionals, such as a primary care physician, specialist, physical therapist, and other healthcare professionals. (Do not include the employer’s occupational physician.)

Healthcare professionals……

- finds it odd that I can do much more on some days than on other days
- thinks I should be tougher.
- takes me seriously.
- gives me unhelpful advice.
- understands the consequences of my health problems or illness.
- makes me feel like I’m exaggerator.
- thinks I can work more than I do.
- gives me the chance to talk about what is on my mind.

**Section 4**: Work environment, such as coworkers and supervisors. (Do not include the employer’s occupational physician.)

If you have not been engaged in any paid or unpaid work in the past year, skip this section.

People at work……

- finds it odd that I can do much more on some days than on other days
- thinks I should be tougher.
- takes me seriously.
- gives me unhelpful advice.
- understands the consequences of my health problems or illness.
- makes me feel like I’m exaggerator.
- thinks I can work more than I do.
- gives me the chance to talk about what is on my mind.

**Section 5:** Social services, your employer’s occupational physician, vocational rehabilitation staff, the employment agency and other government agencies, home care services, public authorities, and health insurance providers.

If you haven’t been in contact with these organizations, you can skip this section.

Vocational rehabilitation staff and government agencies...

- finds it odd that I can do much more on some days than on other days
- thinks I should be tougher.
- takes me seriously.
- gives me unhelpful advice.
- understands the consequences of my health problems or illness.
- makes me feel like I’m exaggerator.
- thinks I can work more than I do.
- gives me the chance to talk about what is on my mind.

____________________________________________________________________

5. ***Category: Perceived Social Support***

1. The Internet can be used to connect with other people online through social networks such as Facebook or Twitter. These networks are often referred to as “social media.” Have you used the Internet in the past 12 months for any of the following reasons?

Check YES or NO

- To visit a social network, such as Facebook or LinkedIn?
- To share health information with others on a social network like Facebook or Twitter?
- To write entries in an online diary or blog?
- To get information or exchange ideas in an online forum or support group for people with similar health issues?
- To watch a video on a health-related topic on YouTube?

2. People sometimes turn to others for companionship, help, or other forms of support. How often do you have access to the following types of support when you need them? Please select one answer for each line. Options: Never, Rarely, Sometimes, Most of the time, Always.

- Someone you can count on to listen to you when you need to talk
- Someone to share your most private worries and fears with
- Someone who understands your problems
- Someone you can confide in or talk to about yourself or your problems
