# Supplementary material for: Medical invalidation is associated with structural barriers in postacute immune mediated syndromes based on patient perspectives in Germany
Source: Sci Rep. 2026 Aug 25;16:26687. doi: 10.1038/s41598-026-67883-2 (PMC13507045; doi:10.1038/s41598-026-67883-2)
Supplement: Supplementary file 2 [file 41598_2026_67883_MOESM2_ESM.docx]

# Appendix 2-10 of the Manuscript “Medical invalidation is associated with structural barriers in postacute immune mediated syndromes based on patient perspectives in Germany“

**Appendix 2 - Recruitment data**

| **Type of recruitment** | | **Number** |
| --- | --- | --- |
| Email contact details from previous studies | | 50 |
| Self-help groups | | 147 |
| Social media | Facebook | 7 |
|  | Instagram | 12 |

**Appendix 3 - Flow Chart**

**Appendix 4 - Career change due to diagnosis**

**Appendix 5 - Specialization of diagnosis (in %)**

Visited diagnostic specialist practices and outpatient clinics

|  | LC/PC | ME/CFS | PACVS |
| --- | --- | --- | --- |
| Post-Covid-Ambulance | 12.1 | 12.2 | 2.3 |
| Post-Vac-clinic | 0 | 0 | 4.7 |
| Family Medicine | 40 | 37.4 | 30.2 |
| Neurology | 24.2 | 27.6 | 32.6 |
| Pulmonology | 9.5 | 5.9 | 7 |
| Immunology | 1.6 | 4.7 | 11.6 |
| Internal medicine | 14.2 | 16.5 | 9.3 |
| Cardiology/Angiology | 5.8 | 5.9 | 37.2 |
| Others | 30.9 | 23 | 53.2 |

**Appendix 6 –** **All Visited diagnostic specialist practices and outpatient clinics**

| **Institutes** | **N (%)** |
| --- | --- |
| Post-Vac Clinic | 2 (0,3) |
| Rehabilitation Clinic | 24 (4,2) |
| Post-COVID Clinic | 55 (9,5) |
| Fatigue Clinic | 3 (0,5) |
| Coagulation Clinic | 1 (0,2) |
| Infectious Diseases Clinic | 1 (0,2) |
| Immunodeficiency Clinic / Medical Immunology | 3 (0,5) |
| Center for Rare Diseases | 2 (0,3) |
| Center for Naturopathy | 1 (0,2) |
| Memory Clinic | 2 (0,3) |
| ME Research Institute | 2 (0,3) |
| **Specialties** |  |
| general practitioner | 184 (31,9) |
| Neurology | 130 (22,5) |
| Pulmonology | 36 (6,2) |
| Ear, Nose and Throat (ENT) | 2 (0,3) |
| Internal Medicine | 73 (12,7) |
| Nephrology | 2 (0,2) |
| Rheumatology | 4 (0,7) |
| Immunology | 20 (3,5) |
| Angiology | 10 (1,7) |
| Infectious Diseases | 11 (1,9) |
| (Pediatric) Cardiology | 32 (5,5) |
| (Neuro)psychology | 7 (1,2) |
| Environmental Medicine | 3 (0,5) |
| Sports Medicine | 6 (1) |
| Speech-Language Pathology | 1 (0,2) |
| Hematology | 1 (0,2) |
| Endocrinology | 2 (0,3) |
| Emergency Medicine / Critical Care Medicine | 2 (0,3) |
| Palliative Medicine | 1 (0,2) |
| Vascular Surgery | 1 (0,2) |
| Anesthesiology | 5 (0,9) |
| Traditional Chinese Medicine | 1 (0,2) |
| Pain Medicine | 5 (0,9) |
| Orthopedics | 2 (0,3) |
| Urology | 1 (0,2) |
| Psychiatry | 28 (4,9) |
| Gastroenterology | 1 (0,2) |
| Dermatology and Venereology | 1 (0,2) |
| Ophthalmology | 2 (0,3) |
| Integrative / Functional Medicine | 4 (0,7) |
| Physical and Rehabilitation Medicine | 5 (0,9) |
| Nutritional Medicine | 1 (0,2) |
| Allergology | 1 (0,2) |
| Pediatrics | 1 (0,2) |
| Neurosurgery | 1 (0,2) |
| Trauma Surgery | 1 (0,2) |

**Appendix 7 -** Visited diagnostic specialist: Cardiology and Immunology

| Field of study | Immunology | Cardiology |
| --- | --- | --- |
| LC/PC & ME/CFS | X² 3.27  (0.07), C 0.08 | X² 1.8 (1.75), C 0.06 |
| LC/PC & PACVS | Fisher p 0.017 | X² 14.5 (<0.001)***, C 0.226 |
| ME/CFS & PACVS | Fisher p 0.208 | Fisher p < 0.001*** |

**Note:** *A comparison of the three diagnostic groups revealed significant differences for the specialties of cardiology and immunology. Chi-square tests and Fisher's exact tests were performed for further examination. The results indicate that cardiology practices were consulted significantly more often by PACVS patients than by patients with LC/PC or ME/CFS. . * p ≤ 0,05, ** p ≤ 0,01, *** p ≤ 0,001.*

**Appendix 8 - Invalidation (3*I) dimensions discounting and lack of understanding**

| Source | LC/PC  M±SD | | ME/CFS  M±SD | | PACVS  M±SD | | Welch-F (p) | |
| --- | --- | --- | --- | --- | --- | --- | --- | --- |
|  | *1 | *2 | *1 | *2 | *1 | *2 | *1 | *2 |
| Partner | 1,96±0,9 | 1,9±0,98 | 2±1 | 2,1±1,1 | 1,95±  0,87 | 2,1±1,07 | 0,23 (0,8) | 1,1 (0,33) |
| Family | 2,6± 1 | 2,7±1,1 | 2,5±1 | 2,6±1,1 | 2,1±  0,9 | 2,7±1 | 4,71 (0,01)** | 0,15 (0,85) |
| Medical staff | 2,68±1 | 2,9±1,02 | 3±1,1 | 3,11±1,04 | 3±1 | 3,24±0,93 | 3 (0,055) | 1,8 (0,162) |
| Work | 3,15±  1,2 | 3,45±1,07 | 3,1 ±  1,1 | 3,55±1,03 | 3,1±  1,06 | 3,36±1,13 | 0,08 (0,91) | 0,48 (0,61) |
| Authority | 3,1±1,1 | 3,4±1,1 | 3,4±1,2 | 3,8±1 | 3,4±0,9 | 4±1 | 3,21 (0,044)* | 5,5 (0,005)** |

Note: Mean scores (M) and standard deviations (SD) for the invalidation (3*I) dimensions discounting and lack of understanding across different sources (partner, family, medical staff, workplace, and authorities) and diagnostic groups (Long COVID, ME/CFS, and post-vaccination syndrome). Group differences were analyzed using Welch’s ANOVA. *1 = discounting; *2 = lack of understanding.*1: Discounting, *2: Lack of Understanding, ** p ≤ 0,05, ** p ≤ 0,01, *** p ≤ 0,001.*

**Appendix 9 - Correlations between invalidation by medical staff and treatment experience**

To assess the convergent validity of 3*I with regard to medical staff, correlations were examined with the following theoretically related variables: a) whether the patient’s symptoms were taken seriously, b) whether all symptoms were considered in the treatment process, and c) whether the symptoms were downplayed or attributed to psychological factors.

Significant correlations were found between perceived invalidation by medical staff and various aspects of the treatment experience. A lower feeling of being taken seriously with one's own complaints was associated with higher scores of overall medical invalidation (Spearman's rₛ = 0.52, p < 0.001). Similarly, there was a significant correlation between reports of symptoms being downplayed or psychologized and higher invalidation scores (r = −0.32, p < 0.001). Failure to take all symptoms into account in the treatment plan was also associated with higher medical invalidation scores (r = 0.32, p < 0.001). Detailed correlation coefficients for the Lack of Understanding and Discounting subscales can be found in the Table:

| **Complaints taken seriously** |  |
| --- | --- |
| Total | rₛ = 0.52, p < 0.001*** |
| Discounting | rₛ = 0.48, p < .001*** |
| Lack of understanding | rₛ = 0.48, p < .001*** |
| **Symptoms downplayed** |  |
| Total | r = -0.32, p < 0.001*** |
| Discounting | r = −0.31, p < 0.001*** |
| Lack of understanding | r = −0.27, p < 0.001*** |
| **Consideration of symptoms** |  |
| Total | r = 0.32, p < 0.001*** |
| Discounting | r = 0.29 p < .001*** |
| Lack of understanding | r = 0.31 p < .001*** |

***Note:*** *Correlations between perceived invalidation by medical personnel and aspects of medical treatment, including whether complaints were taken seriously, whether symptoms were downplayed, and whether symptoms were considered during treatment. Values represent correlation coefficients (Pearson’s r or Spearman’s rₛ) with corresponding p-values. Invalidation is reported for the total score and the subscales discounting and lack of understanding of the 3*I. * p ≤ 0,05, ** p ≤ 0,01, *** p ≤ 0,001.*

**Appendix 10 - Correlations for the individual diagnostic groups between perceived invalidation by medical personel and aspects of medical treatment**

|  | LC/PC | ME/CFS | PACVS |
| --- | --- | --- | --- |
| **Complaints taken seriously**  ( rₛ = 0.52, p < 0.001) |  | | |
| total | r 0,46 p <0,001 | r 0,6 p <0,001 | r 0,5 p 0,16 |
| Lack of Understanding | r 0,44 p <0,001 | 0,57 p <0,001 | r 0,153 p 0,22 |
| Discounting | r 0,4p < 0,001 | r 0,54 p <0,001 | r 0,57 p < 0,001*** |
| **Symptoms downplayed**  r = -0.32, p < 0.001 |  |  |  |
| total | r -0,28 p <0,001 | r -0,36 p <0,001 | r -0,17 p 0,16 |
| Lack of Understanding | r -0,21 p 0,001 | -0,33 p <0,001 | 0,15 p 0,9 |
| Discounting | r -0,28 p < 0,001 | r -0,35 p <0,001 | r -0,24 p 0,056 |
| **Consideration of symptoms**  (r = 0.32, p < 0.001) |  |  |  |
| total | r0,25 p <0,001 | r 0,284 p <0,001 | r 0,26 p 0,035* |
| Lack of Understanding | r0,27 p <0,001 | 0,388 p <0,001 | r 0,054 p 0,668 |
| Discounting | 0,215 p 0,002 | r0,34 p <0,001 | r 0,318 p 0,01** |

***Note:*** *Correlations for the individual diagnostic groups between perceived invalidation by medical personnel and aspects of medical treatment, including whether complaints were taken seriously, whether symptoms were downplayed, and whether symptoms were considered during treatment. Values represent correlation coefficients (Pearson’s r or Spearman’s rₛ) with corresponding p-values. Invalidation is reported for the total score and the subscales discounting and lack of understanding of the Illness Invalidation Inventory (3*I). * p ≤ 0,05, ** p ≤ 0,01, *** p ≤ 0,001.*
